# Unique electrocardiographic pattern “w” wave in lead I of idiopathic ventricular arrhythmias arising from the distal great cardiac vein

**DOI:** 10.1186/s12872-019-1064-9

**Published:** 2019-04-15

**Authors:** Teng Li, Qiong Xu, Xian-zhang Zhan, Yu-mei Xue, Hong-tao Liao, Yi-fu Li, Konstantinos P. Letsas, Shu-lin Wu

**Affiliations:** 1 Arrhythmia Department, Fuwai Hospital Chinese Academy of Medical Sciences, Shenzhen, 518057 Guangdong China; 2 Cardiovascular Department, Guangdong Cardiovascular Institute, Guangdong General Hospital & Guangdong Academy of Medical Sciences, Guangzhou, 510010 Guangdong China; 3 Second Department of Cardiology, Laboratory of Cardiac Electrophysiology, Evangelismos General Hospital of Athens, Athens, Greece

**Keywords:** Idiopathic, Ventricular arrhythmias, Great cardiac vein, Catheter ablation

## Abstract

**Background:**

The ECG characteristics of the distal coronary venous system ventricular arrhythmias (VAs) share common features with VAs arising from the aortic cusps or the endocardial left ventricular outflow tract (LVOT) beneath the cusps. The purpose of this study was to identify specific electrocardiographic and electrophysiological characteristics of VAs originating from the distal great cardiac vein (GCV).

**Methods:**

Based on the successful ablation site, patients with idiopathic VAs from the distal GCV, left coronary cusp (LCC) or the subvalvular left ventricular outflow tract (LVOT) area were included in the present study.

**Results:**

The final population consisted of 39 patients (35 males, mean age 51 ± 23 years). All VAs displayed a right bundle branch block (RBBB) morphology with inferior axis. Among these patients, 15 were successfully ablated at the GCV, 15 at the LCC and 9 at the subvalvular region. A “w” pattern in lead I was present in 12 out of 15 (80%) VAs originating from the distal GCV compared to none of VAs arising from the other two sites (*p* < 0.01). VAs with a GCV origin exhibited more commonly increased intrinsicoid deflection time, higher maximum deflection index and wider QRS duration compared to LCC and subvalvular sites (*p* < 0.05). Acceptable pace mapping at the successful ablation site was achieved in 10 patients. After an average of 36 ± 24 months follow up, 14 (93.3%) patients were free from VAs recurrence.

**Conclusion:**

A “w” pattern in lead I may distinguish distal GCV VAs from VAs arising from the LCC or the subvalvular region.

**Electronic supplementary material:**

The online version of this article (10.1186/s12872-019-1064-9) contains supplementary material, which is available to authorized users.

## Background

Idiopathic ventricular arrhythmias (VAs) can arise from the left ventricular (LV) endocardium and epicardium. The incidence of an epicardial origin may be as high as 9% [1]. The coronary venous system provides an alternative route to target epicardial VAs [2–7]. The ECG characteristics of the distal coronary venous system VAs share common features with VAs arising from the aortic cusps or the endocardial left ventricular outflow tract (LVOT) beneath the cusps [2, 8–11]. Pre-interventional ECG screening of idiopathic VAs for possible epicardial origin is crucial for a targeted ablation. In this study, the ECG and electrophysiological characteristics of VAs originating from the distal great cardiac vein (GCV) were compared with VAs successfully ablated from the left coronary cusp (LCC) or the subvalvular LVOT region.

## Methods

### Study population

In this observational study, a total of 980 patients underwent radiofrequency catheter ablation (RFCA) of symptomatic idiopathic VAs at the Guang-dong General Hospital between January 2012 and August 2014. All patients were refractory to one or two antiarrhythmic drugs. Structural heart disease was ruled out in all patients by means of transthoracic echocardiography, exercise stress test or coronary angiography (CAG) and cardiac magnetic resonance imaging in selected cases. Based on the successful ablation site, patients with VAs from the distal GCV, LCC or the subvalvular LVOT area were included in the present study. All patients provided written informed consent before the procedure, and the study was approved by the institutional review board of Guang-dong General Hospital.

### ECG analysis

The 12-lead ECGs were analyzed at a paper speed of 100 mm/s, and signals were amplified at 10 mm/mv. ECG analysis was focused on the following parameters: i. QRS morphology including bundle-branch block pattern and axis deviation; ii. QRS duration; iii. R wave amplitude in leads II and III; iv. R wave amplitude ratio in leads III to II (III/II); v. QS wave amplitude in leads aVL and aVR; vi. QS wave amplitude ratio in leads aVL to aVR (aVL/aVR); vii. The presence of pseudo-delta wave [12]; viii. Intrinsicoid deflection time (IDT) defined as the interval measured from the earliest ventricular activation to the peak of the R wave in V2; ix. Maximum deflection index (MDI) defined as the interval measured from the beginning of the QRS complex to the maximum deflection in the precordial leads divided by the QRS duration [1]; and ix. The presence of a notch at downslope of an initial q wave resembling a “w” pattern in lead I.

### Electrophysiological study and ablation

The electrophysiological study (EPS) was performed with the patients in a fasting non-sedated state. Antiarrhythmic drugs were discontinued for at least five half-lives prior to the study. A 3.5-mm tip irrigated ablation catheter (Navi-star, Biosense Webster, Diamond Bar, CA, USA, or Cool Path, IBI, St. Jude Medical, Irvine, CA, USA) compatible with a three-dimensional mapping system (CARTO3, Biosense Webster, Inc. or Ensite NavX Velocity, St. Jude Medical) was advanced via percutaneous access through a femoral artery to the aortic root and LVOT region for mapping and ablation. An additional long sheath was placed in the right femoral vein to access the coronary sinus (CS) for epicardial mapping and ablation, if necessary. A diagnostic catheter with 10 electrodes (Biosense Webster, Inc. or St. Jude Medical) was introduced into the CS as deeply as possible to map the GCV.

**Fig. 1:**
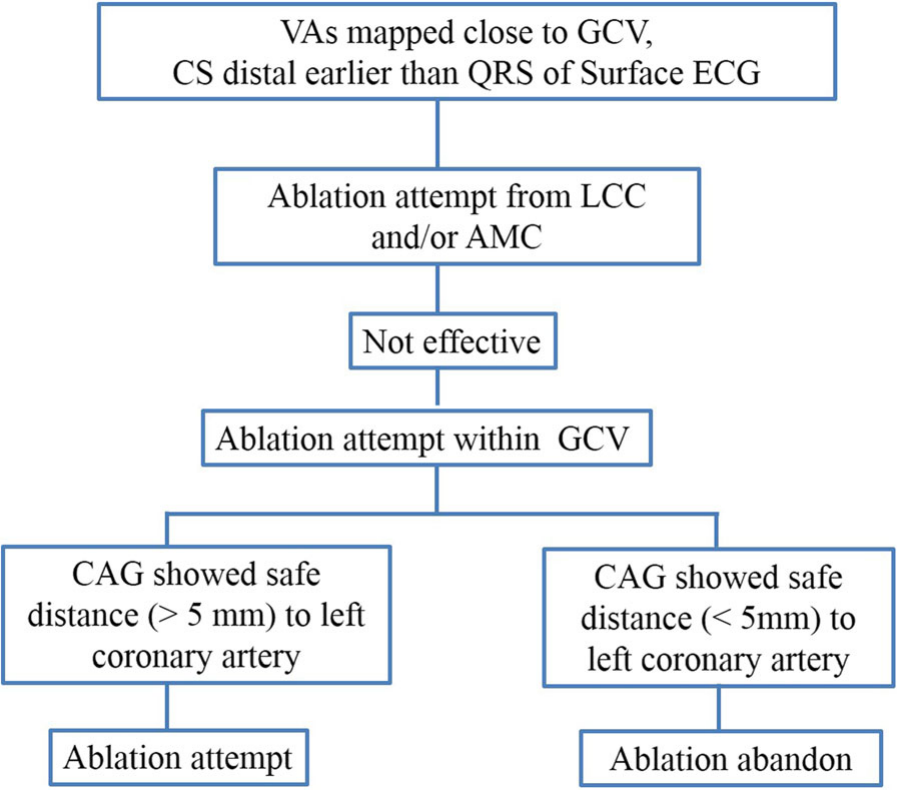
Flowchart displaying the ablation strategy. VAs, ventricular arrhythmias; GCV, great cardiac vein; CS, coronary sinus; LCC, left coronary cusp; AMC, aortomitral continuity; CAG, Coronary angiography

Activation times were measured from the onset of the earliest local bipolar electrogram to the earliest onset of the QRS complex in any of the 12 ECG leads. Pace mapping was additionally performed at the same site. If clinical arrhythmias failed to occur spontaneously, programmed ventricular stimulation was performed. If VAs were not inducible at baseline, intravenous isoproterenol infusion (2 to 5 μg/min) was administered to provoke clinical arrhythmias. Radiofrequency (RF) energy applications (30 W, temperature limit of 43°Cand irrigation rate of 17 ml/min) were stopped if the VAs did not terminate within 20 s. Ablation was not recommended to perform first in the GCV to avoid the risk of injury to coronary artery even the activation of CS catheter preceded the QRS onset by more than 20 ms. If ablation within the aortic cusps or endocardial at the LVOT failed to eliminate the VAs, then additional mapping was performed within the coronary venous system. If the earliest activation within the GCV preceded the QRS onset by more than 20 ms and pacing from that site produced an acceptable QRS match (> 11/12 leads), ablation within the GCV was attempted (Fig. 1). Prior to ablation within the GCV, CAG was performed to access the potential risk of coronary artery damage. RF energy applications were never delivered when the distance between the earliest activation site and an epicardial coronary artery was less than 5 mm. RF energy was applied at the earliest activation site in the GCV using an irrigated ablation catheter with a power of 20 W, temperature limit of 43 °C and an irrigation rate of 30 ml/min. The RF power was titrated to a maximal of 30 W. The end-point of the catheter ablation was the elimination and non-inducibility of VAs during an isoproterenol infusion and burst pacing from the right ventricle (to a cycle length as short as 300 ms). Following GCV ablation, CAG was repeated to ensure that there was no evidence of injury to the coronary artery. The site of origin of VAs was determined based on successful elimination with RF energy application.

### Follow-up

After the procedure, continuous ECG monitoring was performed for 24 h. Antiarrhythmic drugs were discontinued in the absence of arrhythmia recurrence. Patients were evaluated every 3 months in an outpatient clinic. 24-h Holter monitoring was carried out in every visit. If patients report any chest discomfort symptoms must undergo treadmill exercise testing or event CAG again to eliminate the possibility of coronary artery damage.

### Statistical analysis

Continuous variables (expressed as mean ± SD) were compared with Student t test (or Wilcoxon when necessary). Categorical variables were compared with chi-squared or Fisher exact test, as appropriate. A probability value < 0.05 was considered statistically significant.

## Results

### Patient characteristics

The final population consisted of 39 patients (35 males, mean age 51 ± 23 years). Among these patients, 15 were successfully ablated at the distal GCV, 15 at the LCC and 9 at the subvalvular region (below the LCC). The baseline characteristics of the study cohort are depicted in Table 1. None of the patients displayed a family history of sudden cardiac death or a history of previous cardiovascular disease and decreased left ventricular ejection fraction. None of the patients had failed previous ablation procedures. The dominant arrhythmia was symptomatic VAs in all GCV group patients with high burden (23 ± 6%).

**Table 1:** Differences in baseline patient characteristics and VAs electrocardiographic morphologies

Characteristic	GCV group (*n* = 15)	LCC group (*n* = 15)	Subvalvular region group (*n* = 9)	*P* value^*^	*P* value^#^
Male, n (%)	14(93%)	13(87%)	8(89%)		
Age (years)	51 ± 23	48 ± 21	50 ± 14	0.71	0.81
VAs burden (%)	23 ± 6	20 ± 6	23 ± 5	0.13	0.85
LVEF (%)	64.8 ± 4.3	63.2 ± 4.8	62.0 ± 5.1	0.34	0.17
Structural heart disease (%)	0	0	0		
Limb leads					
R-wave amplitude in lead II (mV)	1.45 ± 0.46	2.04 ± 0.36	2.28 ± 0.20	< 0.05	< 0.05
R-wave amplitude in lead III(mV)	1.74 ± 0.53	2.22 ± 0.29	2.72 ± 0.30	< 0.05	< 0.05
III/II R-wave amplitude ratio	1.21 ± 0.12	1.11 ± 0.09	1.19 ± 0.07	0.06	0.65
Q-wave amplitude in lead aVR(mV)	0.65 ± 0.22	1.26 ± 0.23	0.97 ± 0.10	< 0.05	< 0.05
Q-wave amplitude in lead aVL(mV)	1.05 ± 0.31	1.32 ± 0.12	1.58 ± 0.19	< 0.05	< 0.05
aVL/aVR Q-wave amplitude ratio	1.72 ± 0.39	1.51 ± 0.2	1.67 ± 0.43	0.07	0.77
Duration (ms)					
QRS duration	172 ± 19	148 ± 5	152 ± 6	< 0.05	< 0.05
IDT	110 ± 16	96 ± 9	93 ± 8	< 0.05	< 0.05
Delta wave (ms)	52 ± 11	46 ± 7	48 ± 8	0.86	0.16
MDI	0.64 ± 0.05	0.60 ± 0.04	0.61 ± 0.02	< 0.05	< 0.05

*VAs* ventricular arrhythmias, *GCV* great cardiac vein, *LCC* left coronary cusp, *IDT* intrinsicoid deflection time, *MDI* maximum deflection index. *Comparison between GCV and LCC group, ^*#*^Comparison between GCV and Subvalvular region group

### ECG characteristics

**Fig. 2:**
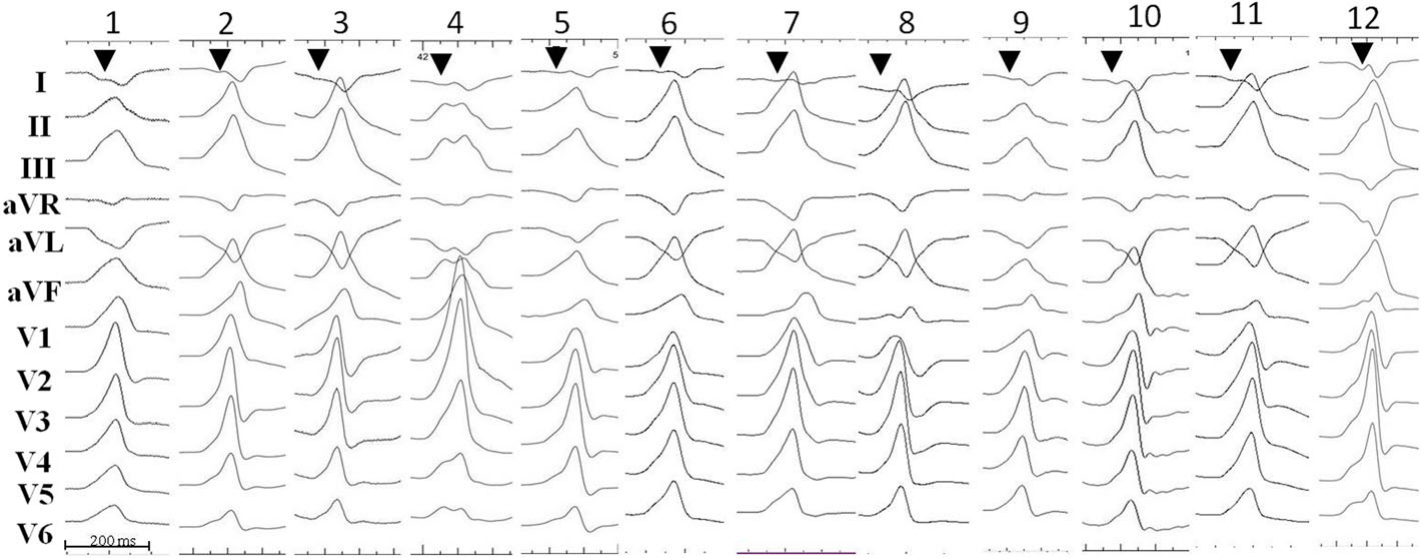
Twelve-lead ECG of the ventricular arrhythmias arising from the distal great cardiac vein are shown. The presence of a notch at downslope of an initial q wave resembling a “w” pattern in lead I is shown (arrow)

The ECG characteristics of VAs with to respect to successful site of ablation are shown in Table 1. All GCV VAs displayed a right bundle branch block (RBBB) pattern with inferior axis (Fig. 2). In particular, GCV VAs exhibited positive QRS forces in all precordial leads with no S waves in leads V5 and V6. An Rs pattern in leads V2-V4 was evident in all but two patients. VAs with a GCV origin exhibited more commonly a positive QRS complex concordance in all precordial leads, dominant negative waves in leads I and aVL, increased IDT, higher MDI and wider QRS duration (172 ± 19 ms) compared to LCC and subvalvular sites (*p* < 0.05). There were no significant differences of the (III/II) R wave amplitude ratio and the (aVL/aVR) QS wave amplitude ratio between GCV group and LCC or subvalvular groups (*p* > 0.05). A trend towards a higher (aVL/aVR) QS wave amplitude ratio was seen in VAs originating from the GCV compared to VAs arising from the LCC (1.72 ± 0.39 vs. 1.51 ± 0.2, p < 0.05).

A “w” pattern in lead I (Fig. 2) was present in 12 out of 15 (80%) VAs originating from the GCV compared to VAs arising from the other two sites (*P* < 0.01). An rS pattern in lead I was evident in the remaining 3 GCV patients. A QS or rS pattern in lead I was present in patients with LCC and endocardial LVOT origins.

### Mapping and ablation

**Fig. 3:**
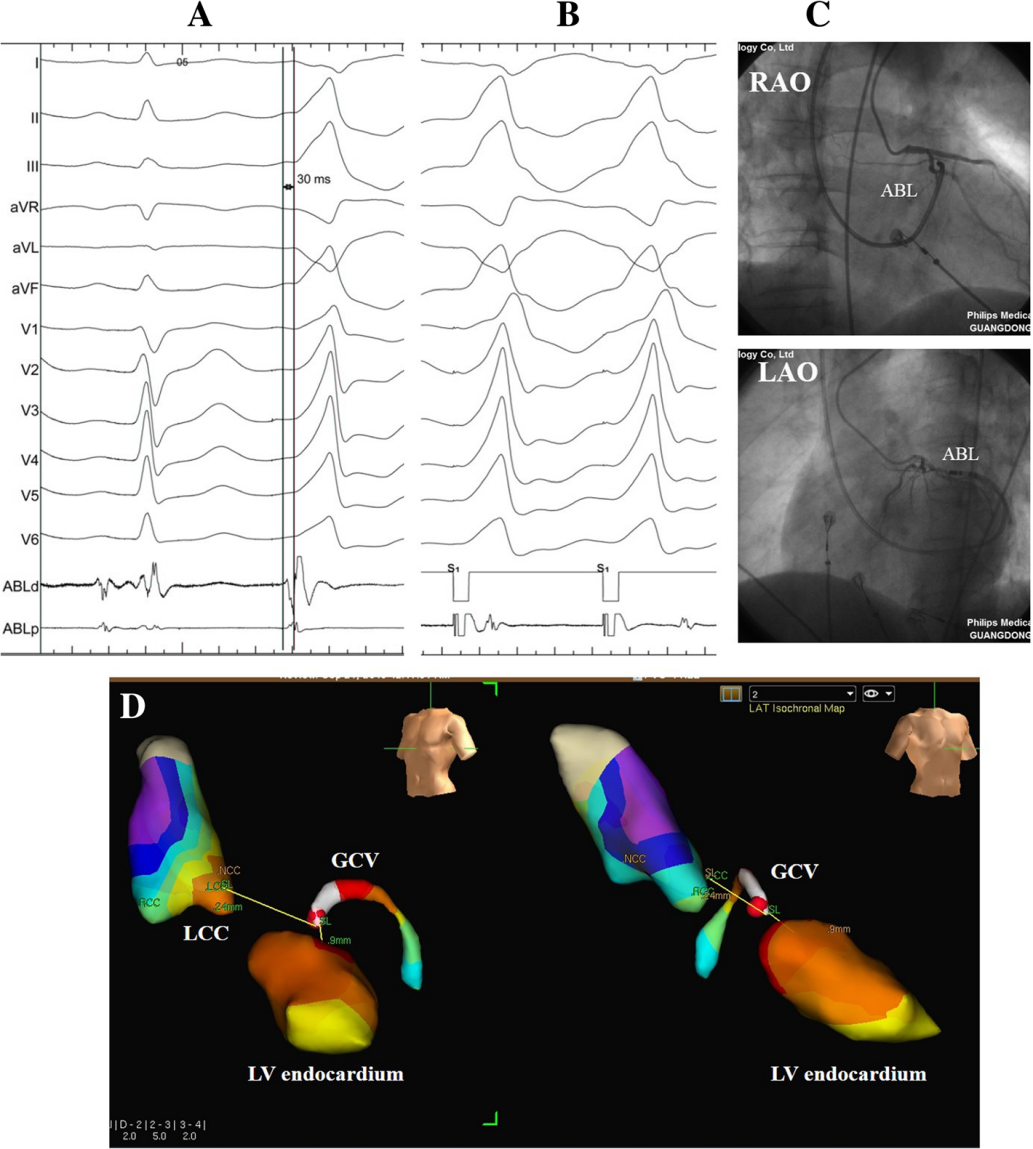
Example of a successful ablation of premature ventricular contractions originating from the distal GCV. **a** Activation time at the GCV (30 ms before QRS onset). **b** Pace-map at the GCV demonstrating an excellent match. **c** Corresponding fluoroscopic view (RAO 30°and LAO 45°) of the ablation catheter (ABL) positions during coronary angiography of the left coronary artery (Additional files 3 and 4: Video S1 and S2). The ablation catheter tip is ≥5 mm distant from any coronary artery. **d** The anatomical distance from the earliest activation site at the distal GCV to the closest anatomical point at the LCC and the LV endocardium were 24 mm and 9 mm, respectively. ABL d (p), the distal and proximal electrode pairs of the ablation catheter; GCV, great cardiac vein; CS, coronary sinus; RAO, right anterior oblique projection; LAO, left anterior oblique projection; LCC, left coronary cusp, LV, left ventricle

**Fig. 4:**
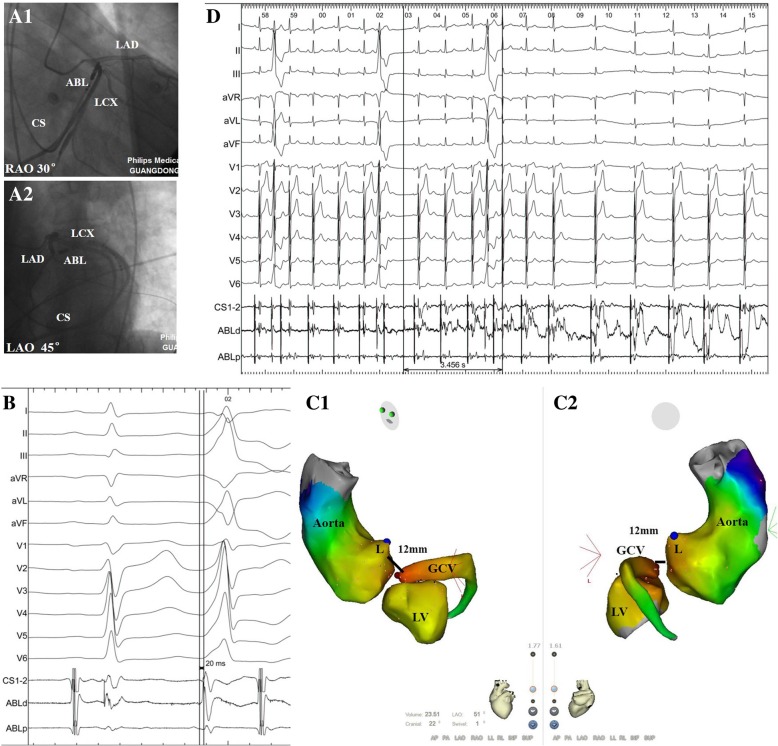
Fluoroscopy, activation, and 3-dimensional mapping in a 55-year-old man with successful ablation of premature ventricular contractions at the distal of great coronary vein (GCV). **a** (A1 and A2): Corresponding fluoroscopic views (RAO 30°and LAO 45°) of the ablation catheter (ABL) at the successful ablation site at the distal of GCV during left coronary angiography. **b** surface ECG and intracardiac recordings from the mapping catheter (ABL) at the site of earliest ventricular activation at the distal GCV. Note that the local potential precedes the QRS by 20 ms during ventricular extrasystoles. **c** (C1 and C2): Electroanatomic maps of the same patient demonstrating the successful ablation point (red tag) at the distal GCV. **d** Termination of PVCs within 4 s of radiofrequency energy application at the distal GCV. Blue point indicates left main coronary artery. L, left coronary cusp. CS, coronary sinus catheter; LAO, left anterior oblique; LAD, left anterior descending artery; LAO, left anterior oblique; LCX, left circumflex artery

Access to the left ventricle for mapping and ablation was performed retrogradely. Among the 15 patients with GCV VAs (Fig. 3), mapping and ablation of proceeded via right femoral vein in 13 patients at GCV and via the left subclavian vein in two patients. The targeted potential at the final ablation site preceded QRS complex onset of clinical VAs by 25 ± 5 ms (Figs. 3 and 4). Of these 15 patients, Pace mapping at the successful ablation site in GCV was obtained in only 10 patients.

During sinus rhythm, a clear atrial potential was always observed before the ventricular activation (Fig. 4). During VAs, a pre-potential was observed at the initial ventricular activation in 5 of 15 patients (33%). 12-lead ECG and local electrogram of successful ablation site in three representative cases of the distal GCV, LCC, and subvalvular LVOT were compared (Additional file 1: Figure S1). Mean time to clinical VAs disappearance was 6 ± 3 s. Among 15 patients with GCV VAs, impedance in the epicardium was much higher than LV endocardium (187 ± 9 Ω vs. 117 ± 9 Ω, *P* < 0.05). The VAs was successfully abolished with a mean of 2.3 ± 1.4 RF applications. No ST segment or T wave changes were observed during ablation in any of the 15 patients. Procedure time was 127 ± 57 min with fluoroscopy time of 12.3 ± 6.4 min. The distance between the successful ablation site in the GCV and the best (but failed) site in the endocardium was 14 ± 4 mm.

In the initial study population, 24 patients exhibited the earliest activation site at the distal GCV. Nine of these patients were excluded from the current analysis due to failure to advance the ablation catheter at the distal GCV (*n* = 4), failure to eliminate the VAs with RF applications (*n* = 3) and abandoned ablation because of the close proximity to a major coronary artery (*n* = 2). A schematic diagram shows the sites of origin of 15 GCV-VAs determined by the CAG of the left coronary artery and three-dimensional mapping (Additional file 2: Figure S2).

### Follow-up

After an average of 36 ± 24 months follow up, 14 (93.3%) patients were free from VAs recurrence. No complications occurred immediately after the procedure or during follow up. None of the patients reported chest pain or discomfort symptoms at follow-up and therefore subsequent CAG was not repeated.

## Discussion

### Major findings

The main findings of the present study are the following: i. a “w” pattern in lead I distinguish a GCV origin from LCC or endocardial origins; ii. GCV VAs display an increased IDT and MDI compared to LCC or endocardial sites.

### ECG morphology criteria

The ECG characteristics of VAs originating from the distal GCV varies. In the proximal segment, VAs display a RBBB morphology and in the distal segment as well in the anterior interventricular vein a LBBB morphology [2, 8, 11]. In our series, all cases displayed a RBBB, and therefore the proximal segment of distal GCV appears to be the accurate site of origin. Previous studies addressing the QRS morphology in lead I have demonstrated more commonly an rS and less commonly a QS pattern [2, 8, 11].

In this study, the presence of a notch at downslope of the initial q wave resembling a “w” pattern in lead I was present in 80% of VAs originating from the distal GCV compared to none of VAs arising from the LCC and the endocardial LVOT areas. GCV VAs are mostly located at epicardium of superior-lateral LV. The presence of initial negative forces (q wave) in lead I is indicative of an initial rightward activation of the LV base from an epicardial origin. VAs originating from the epicardium showed a q wave in lead I as initial activation which means that the net vector of the global activation pattern from left to right and superior to inferior [13]. The presence of a q wave in lead I and absence of q waves in the inferior leads appear to be a very sensitive criterion for identifying an epicardial site of origin [13]. Therefore, a “w” pattern in lead I raises a high degree of suspicion for a distal GCV origin.

A trend towards a higher (aVL/aVR) QS wave amplitude ratio was seen in VAs originating from the GCV compared to VAs arising from the LCC, which is consistent with previous findings [8]. Finally, we showed that distal GCV VAs display an increased MDI and IDT compared to LCC or endocardial sites. A MDI > 0.59 has been previously shown to identify VAs of an epicardial origin [1, 13]. In our study, all patients had a MDI > 0.60 (100% sensitivity). Previous reports with VAs arising from the distal GCV have consistently demonstrated a higher MDI in relation to endocardial sites [2, 14]. Li et al. have demonstrated that pseudo wave time and IDT were significantly longer for VAs of distal GCV origin compared to VAs from the cusps or the endocardium [14].

### Mapping and ablation

In our study, catheter ablation of VAs arising from the distal GCV was highly effective, in the cases where RF applications could be successfully delivered. However, catheter ablation of VAs arising from the distal GCV is challenging. We showed that in a significant number of patients (9/24) certain limitations do not allow catheter ablation. First, it is not always possible to advance the ablation catheter to the distal GCV. Second, the high impedance and/or the limited cooling from the blood flow may lead to ineffective RF applications. Third, the close proximity to the coronary arteries does not permit a safe ablation procedure. Nagashima et al. reported occlusion requiring stenting of a marginal branch of the circumflex artery in 2 who received RF energy at sites 2 mm and 5–7 mm distant from the vessel, respectively [11]. A distance of 5–12 mm between the ablation catheter and the coronary artery may reduce the risk of injury[15].

VAs displayed a RBBB morphology with inferior axis could safely be eliminated in the GCV, but sometimes ablation can be challenging because of the close proximity to the coronary arteries and failure to advance the ablation catheter at the distal GCV. RFCA within GCV may be limited by a high impedance. Potential risks (Coronary vasospasm, coronary artery damage or perforation) of ablation within the GCV are much more than in the endocardium. Repeated CAG and careful titration of irrigated radiofrequency energy for successful ablation without acute complications. Even no complications were reported in this study, we recommend catheter ablation performed within the distal of GCV with strong clinical indications including symptomatic high burden VAs, arrhythmia induced cardiomyopathy and special profession such as pilot and sports athletes. Besides, catheter ablation was performed in the GCV only after attempting to eliminate the VAs in the endocardium has failed.

A few cases of GCV VAs might be eliminated through LVOT endocardium ablation. Because the breakout site of GCV VAs maybe located adjacent LV epicardium, but the true origin maybe located adjacent LV endocardium. So these VAs originating from the GCV could be ablated from the LCC/adjacent LV endocardium. We could not determine the true origin site of the VAs even after they were eliminated. As a result, the site of origin of VAs was determined based on successful elimination in our study.

### Study limitations

This is a single-center observational and retrospectively study including a small number of patients with relatively uncommon VAs. Although none of the patients reported symptoms during follow-up, coronary artery damage in terms of stenosis cannot be excluded. Due to the signal interference of our catheter labs, the unipolar electrogram was very difficult to identify during mapping and ablation, so the data about unipolar electrogram was not available in this study.

## Conclusions

VAs originating from the GCV represents a rare subgroup of idiopathic VAs. Among patients with outflow tract VAs exhibiting a RBBB morphology, inferior axis, and QRS complex predominantly positive in all precordial leads, a “w” pattern in lead I, an increased IDT, a higher MDI and a wider QRS duration predicted a GCV origin compared to LCC or endocardial sites.

## Additional files

Additional file 1: **Figure S1.** Example of three successful ablations of premature ventricular contractions originating from the distal GCV, LCC, and subvalvular LVOT, respectively. A: Activation time (30 ms before QRS onset) at the GCV of case 1 with VAs originated from the distal GCV. B: Activation time (24 ms before QRS onset) at the LCC of case 2 with VAs originated from LCC. C: Activation time (28 ms before QRS onset) beneath LCC of case 3 with VAs originated from the distal GCV. B: Activation time (24 ms before QRS onset) at the LCC of case 2 with VAs originated from subvalvular LVOT. ABL d (p), the distal and proximal electrode pairs of the ablation catheter; GCV, great cardiac vein; LCC, left coronary cusp; LVOT, left ventricular outflow tract. (JPG 162 kb)

Additional file 2: **Figure S2.** A schematic representation of the anatomic distribution of successful ablation sites in the 15 GCV-VAs. Left lateral view of the heart. Black circles represented the successful ablation sites. GCV, great cardiac vein; LAD, left anterior descending; CS, coronary sinus; LV, left ventricle; LAA, left atrial appendage; LSPV, left superior pulmonary vein; LIPV, left inferior pulmonary vein; RV, right ventricle. (JPG 177 kb)

Additional file 3: **Video S1.** Fluoroscopic view (LAO 45°) of the ablation catheter (ABL) positions during coronary angiography of the left coronary artery. (AVI 4868 kb)

Additional file 4: **Video S2.** Fluoroscopic view (RAO 30°) of the ablation catheter (ABL) positions during coronary angiography of the left coronary artery. (AVI 4100 kb)

## Abbreviations

CAG: Coronary angiography
CS: Coronary sinus
EPS: Electrophysiologic study
GCV: Great cardiac vein
IDT: Intrinsicoid deflection time
LCC: Left coronary cusp
LVEF: Left ventricular ejection fraction
LVOT: Left ventricular outflow tract
MDI: Maximum deflection index
PVC: Premature ventricular contraction
RBBB: Right bundle branch block
RF: Radiofrequency
RFCA: Radiofrequency catheter ablation
RV: Right ventricular
VAs: Ventricular arrhythmias
