# Supplementary figures and images for: Unique electrocardiographic pattern “w” wave in lead I of idiopathic ventricular arrhythmias arising from the distal great cardiac vein
Source: BMC Cardiovasc Disord. 2019 Apr 15;19:90. doi: 10.1186/s12872-019-1064-9 (PMC6466655; doi:10.1186/s12872-019-1064-9)

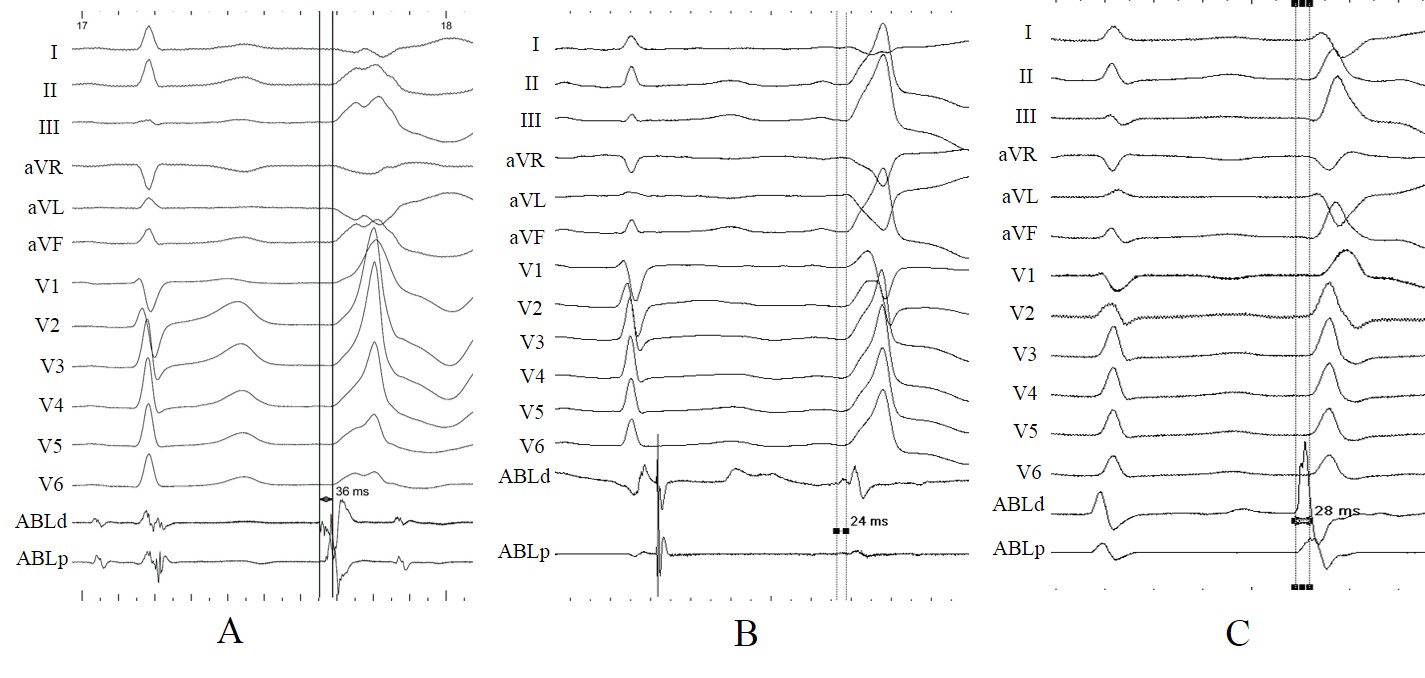

Supplement: Supplementary file 1 — Figure S1. Example of three successful ablations of premature ventricular contractions originating from the distal GCV, LCC, and subvalvular LVOT, respectively. A: Activation time (30 ms before QRS onset) at the GCV of case 1 with VAs originated from the distal GCV. B: Activation time (24 ms before QRS onset) at the LCC of case 2 with VAs originated from LCC. C: Activation time (28 ms before QRS onset) beneath LCC of case 3 with VAs originated from the distal GCV. B: Activation time (24 ms before QRS onset) at the LCC of case 2 with VAs originated from subvalvular LVOT. ABL d (p), the distal and proximal electrode pairs of the ablation catheter; GCV, great cardiac vein; LCC, left coronary cusp; LVOT, left ventricular outflow tract. (JPG 162 kb) [file 12872_2019_1064_MOESM1_ESM.jpg]

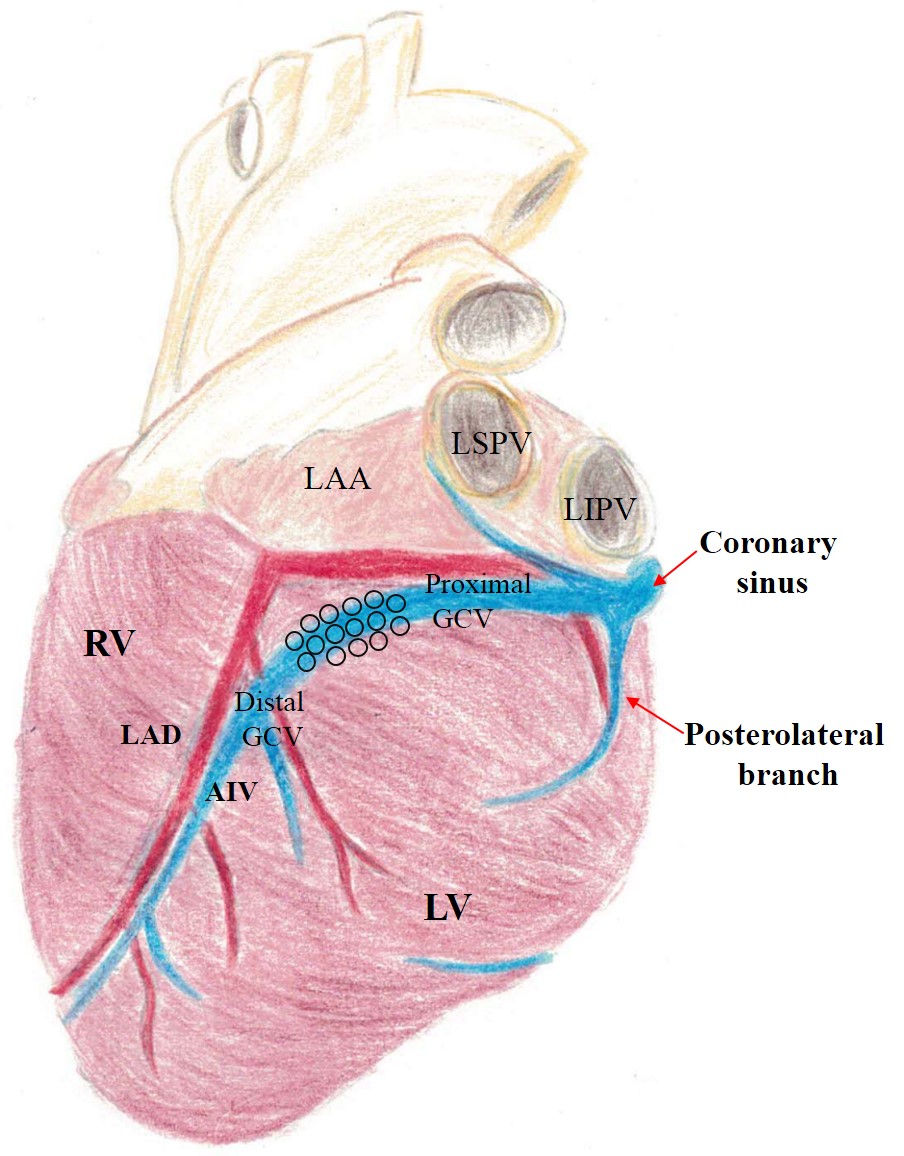

Supplement: Supplementary file 2 — Figure S2. A schematic representation of the anatomic distribution of successful ablation sites in the 15 GCV-VAs. Left lateral view of the heart. Black circles represented the successful ablation sites. GCV, great cardiac vein; LAD, left anterior descending; CS, coronary sinus; LV, left ventricle; LAA, left atrial appendage; LSPV, left superior pulmonary vein; LIPV, left inferior pulmonary vein; RV, right ventricle. (JPG 177 kb) [file 12872_2019_1064_MOESM2_ESM.jpg]
